# Involvement of the tumour necrosis factor receptor system in glioblastoma cell death induced by palbociclib-heptamethine cyanine dye conjugate

**DOI:** 10.1186/s12964-023-01277-z

**Published:** 2024-01-11

**Authors:** Elizabeth Cooper, Caitlin R. M. Oyagawa, Rebecca Johnson, Peter J. Choi, Jena Macapagal Foliaki, Jason Correia, Patrick Schweder, Peter Heppner, Edward Mee, Clinton Turner, Richard Faull, William A. Denny, Mike Dragunow, Jiney Jose, Thomas I-H. Park

**Affiliations:** 1 Auckland Cancer Society Research Centre, School of Medical Sciences, University of Auckland, Private Bag 92019, Auckland, 1142 New Zealand; 2 Department of Pharmacology, The Centre for Brain Research, University of Auckland, Private Bag 92019, Auckland, 1142 New Zealand; 3 Neurosurgery Research Unit, The Centre for Brain Research, University of Auckland, Private Bag 92019, Auckland, 1142 New Zealand; 4 Department of Neurosurgery, Auckland City Hospital, Private Bag 92024, Auckland, 1142 New Zealand; 5 Department of Anatomical Pathology, Auckland City Hospital, 2 Park Road, LabPlus, Auckland, New Zealand

## Abstract

Glioblastoma is the most common and aggressive primary brain tumour in adults. The development of anti-brain cancer agents are challenged by the blood-brain barrier and the resistance conferred by the local tumour microenvironment. Heptamethine cyanine dyes (HMCDs) are a class of near-infrared fluorescence compounds that have recently emerged as promising agents for drug delivery. We conjugated palbociclib, a cyclin-dependent kinase (CDK) 4/6 inhibitor, to an HMCD, MHI-148, and conducted drug activity analysis on primary patient-derived glioblastoma cell lines. In addition to the expected cytostatic activity, our in vitro studies revealed that palbociclib-MHI-148 conjugate resulted in an almost 100-fold increase in cytotoxicity compared to palbociclib alone. This shift of palbociclib from cytostatic to cytotoxic when conjugated to MHI-148 was due to increased DNA damage, as indicated by an increase in γH2AX foci, followed by an increased expression of key extrinsic apoptosis genes, including TP53, TNFR1, TRAIL, FADD and caspase 8. In addition, we observed a time-dependent increase in the cell surface expression of TNFR1, consistent with an observed increase in the secretion TNFα, followed by TNFR1 endocytosis at 48 h. The treatment of patient GBM cells with the palbociclib-MHI-148 conjugate prevented TNFα-induced NFκB translocation, suggesting conjugate-induced TNFR1 signalling favoured the TNFR1-mediated apoptotic response rather than the pro-inflammatory response pathway. Notably, pharmacological inhibition of endocytosis of TNFR1, and siRNA-knockdown of TNFR1 reversed the palbociclib-MHI-148-induced cell death. These results show a novel susceptibility of glioblastoma cells to TNFR1-dependent apoptosis, dependent on inhibition of canonical NFκB signalling using our previously reported palbociclib-HMCD conjugate.

Video Abstract

**Supplementary Information:**

The online version contains supplementary material available at 10.1186/s12964-023-01277-z.

## Introduction

 Glioblastoma is the most common, aggressive, and fatal primary brain tumour in adults, with the global age-standardized rate of primary brain tumours being approximately 6.8 per 100,000 [1]. Relative to glioblastoma’s low incidence, there is a disproportionately higher fatality rate due to its highly aggressive and treatment-resistant nature. The current standard of care is comprised of a rigorous trimodal treatment regimen, including maximal surgical resection, followed by concurrent radiation and temozolomide therapy. However, the five-year survival rate for glioblastoma patients is less than 6%, with a median overall survival time of 12–15 months following diagnosis [2].

Despite significant progress in our understanding of glioblastoma tumourigenesis, advancements in expanding our repertoire of effective therapies have failed to parallel those seen in other cancers. A major hurdle for chemotherapeutic treatments for glioblastoma is the poor bioavailability resulting from the limited blood-brain barrier (BBB) penetration of these agents [3]. The BBB regulates the movement of molecules from the systemic circulation into the brain [3], and with the exception of a few drugs approved for the treatment of brain tumours (e.g., temozolomide), anticancer drugs are generally excluded by this barrier [4, 5]. Improving the delivery of anticancer drugs across the BBB into the brain is, therefore, a critical step in developing an effective chemotherapeutic treatment for brain tumours.

One of the key hallmarks of glioblastoma pathogenesis is alterations in mitogenic signalling that result in the sustained proliferation of the tumour cells, for which inhibitors of cyclin-dependent kinase (CDK) inhibitors may offer beneficial outcomes. CDKs are a family of 13 conserved serine/threonine protein kinases which play essential roles in cell cycle regulation and the maintenance of normal cell proliferation [6]. These kinases are heavily implicated in sustained proliferative activity because of cyclin pathway genomic alterations following malignant transformation. Alterations in cyclin activation genes have been detected in 24% of malignancies; hence, pharmacological targeting of CDKs has emerged as a key focus of cancer research [7].

Currently, three CDK4/6 inhibitors (palbociclib, ribociclib, abemaciclib) have been approved, for the treatment of advanced-stage or metastatic, hormone-receptor-positive, HER2-negative breast cancer [8]. However, the spectrum of CDK inhibitors is broadening, with inhibitors targeting other CDKs currently undergoing clinical trials. 50% of glioblastoma patients have been found to harbour alterations in cyclin D1/CDK4/6-CDKN2A(p16INK4A)-Rb axis; hence, the use of CDK inhibitors in the treatment of glioblastoma warrants further investigation [7].

CDK4 and CDK6 inhibitors bind to the ATP cleft of the target CDK. Palbociclib is specifically active against CDK4/D1, CDK4/D3 and CDK6/D6 [9], where predictive biomarkers of response include cyclin D1 alterations as well as CDKN2A/B inactivation [10]. Although CDK4/6 inhibitors have ample BBB penetration, their retention is limited by efflux through ABCB_1_ and P-gp transporters [11, 12]; hence, clinical trials for glioblastoma have been unsuccessful [13]. However, given the implication of CDK alterations in glioblastoma tumourigenesis [7], CDK inhibitors warrant further investigation.

Heptamethine cyanine dyes (HMCDs) are a class of near-infrared fluorescent (NIRF) compounds that have recently emerged as promising agents for drug delivery with tumour-targeting properties. Their fluorescent and tumour-targeting properties, which were initially explored for imaging neoplasms, can also be harnessed to deliver anti-cancer agents into brain tumours [14–17]. The tumour-targeting properties are attributed to their uptake by isoforms of organic anion transporting polypeptides (OATPs) and albumin receptors, which are overexpressed in cancer tissues [18–21], including glioblastoma [22]. Based on these properties, we resynthesized the HMCD-palbociclib conjugate, hereafter referred to as MHI 148-palbociclib, previously reported by our group [23] to examine its effect and explored the mechanism of action in patient-derived glioblastoma cells.

As described above, despite the centrality of cellular proliferation in glioblastoma tumourigenesis, the clinical activity of CDK4/6 inhibitors has been limited. Although the development of brain-penetrant CDK4/6 inhibitors have been explored, these have yet to be translated to the clinic [24–26]. Critically, where CDK4/6 inhibitors have been successful, such as in hormone-receptor-positive breast cancer [8, 27], recent evidence suggests that the tumour regression observed in these patients cannot simply be due to the cytostatic effects of inhibiting cell proliferation [28–30]. It has become increasingly apparent that kinase inhibitors developed against one target likely have widespread activity amongst other kinases and downstream substrates. However, where this was previously considered a challenge in drug development due to broad substrate toxicity, research efforts have been geared towards understanding the kinome coverage of anti-cancer agents as an opportunity for drug-repurposing and combinatorial treatment [31, 32]. Treatment combinations targeting apoptosis to improve immunotherapy is an approach that is being explored across oncology. Given that the immune system kills tumour cells by induction of apoptosis, [33–35] combining immunotherapy with agents that facilitate the induction of apoptosis would appear a logical treatment regimen for glioblastoma.

Triggering apoptosis in tumour cells by targeting death receptors has been considered an attractive anti-cancer therapy [34, 36, 37]. The majority of death-inducing agents have been reported to activate one of the two molecular pathways, commonly referred to as the extrinsic and intrinsic death pathways [38]. Activation of death receptors of the TNFR family initiates signalling pathways leading either to cell death or survival [39]. TNFα is a pro-inflammatory cytokine whose signalling pathways are linked to both proapoptotic and antiapoptotic responses [39]. Despite TNFα’s ability to induce apoptosis, glioblastoma is reportedly resistant to TNFα-mediated apoptosis [39–41]. Resistance to TNFα-induced apoptosis has been attributed to the activation of NFκB, as blockade of NFκB sensitizes cells to TNFα-induced apoptotic cell death [39–41]. Since CDK4/6 activates NFκB signalling, we have examined whether blockade of CDK4/6 with palbociclib-MHI 148 conjugate, **1**, could activate the endogenous TNFα mediated apoptosis pathway in glioblastoma. We show that patient-derived glioblastoma cells are capable of autocrine/paracrine TNFα-TNFR1 dependent cell death, identifying TNFR1, TNFα and NFκB as key targets in this pathway. As a proof-of-concept, we demonstrate that the inhibition of NFκB by palbociclib-MHI 148 conjugate sensitizes glioblastoma cells to TNFα treatment.

## Methods

### Tissue sources

Primary human glioblastoma tumour samples were isolated and cultured as previously described [42]. Characteristics of glioblastoma cell lines used within this chapter a summarised in Table 1 .

**Table 1:** Characteristics of human neurosurgical biopsy tissue used in experiments

Case number	Age	Sex	Pathology	MGMT status
T069	70	M	glioblastoma	Methylated
T073	55	F	glioblastoma	Unmethylated
T084	69	M	glioblastoma	Unmethylated
T115	67	M	glioblastoma	Unmethylated
T141	61	M	glioblastoma	Unmethylated
T146	60	M	glioblastoma	Methylated

### Establishing the potency of palbociclib-HMCD conjugate, 1, in patient-derived glioblastoma cells and non-tumour neurovascular cells

To establish the potency of **1** on patient-derived glioblastoma cells; five-fold serial dilutions of palbociclib, **1**, MHI-148 and TMZ were prepared between 0.04 nM and 100 µM. The IC_50_ (nM) and the pIC_50_ (-log_10_, IC_50_, M) were determined using EdU as an index of the percentage of proliferating cells. The EC_50_ and the pEC_50_ (-log_10_, EC_50_, M) were determined using Hoechst as an index of the total number of cells. A three-parameter non-linear variable regression curve was fitted using GraphPad Prism using the log (concentration) of each compound versus the relevant measure (IC_50_: Eq. 1, EC_50_: Eq. 2), normalised to the vehicle (0.01% DMSO).

1 $$\varvec{Y}=\varvec{B}\varvec{o}\varvec{t}\varvec{t}\varvec{o}\varvec{m}+\frac{\left(\varvec{T}\varvec{o}\varvec{p}-\varvec{B}\varvec{o}\varvec{t}\varvec{t}\varvec{o}\varvec{m}\right)}{\left(1+ {10}^{\left(\left(\varvec{L}\varvec{o}\varvec{g} {\varvec{I}\varvec{C}}_{50}-\varvec{X}\right)\;\varvec{x}\;\varvec{H}\varvec{i}\varvec{l}\varvec{l}\; \varvec{S}\varvec{l}\varvec{o}\varvec{p}\varvec{e}\right)}\right)}$$

2 $$\varvec{Y}=\varvec{B}\varvec{o}\varvec{t}\varvec{t}\varvec{o}\varvec{m}+\frac{\left(\varvec{T}\varvec{o}\varvec{p}-\varvec{B}\varvec{o}\varvec{t}\varvec{t}\varvec{o}\varvec{m}\right)}{\left(1+ {10}^{\left(\left(\varvec{L}\varvec{o}\varvec{g} {\varvec{E}\varvec{C}}_{50}-\varvec{X}\right)\;\varvec{x}\;\varvec{H}\varvec{i}\varvec{l}\varvec{l}\;\varvec{S}\varvec{l}\varvec{o}\varvec{p}\varvec{e}\right)}\right)}$$

### Establishing synergistic effects of 1 using compuSyn software

To establish the synergistic effect of the palbociclib-MHI 148 conjugate, **1**, with TMZ on patient-derived glioblastoma cells; three-fold serial dilutions of the compounds listed above were prepared between 0.04 nM and 100 µM. Glioblastoma cells were treated with the respective compounds for 96 hs, and the percentage of Hoechst-positive cells were used to determine the synergistic effects with TMZ CompuSyn software was used to establish the combination index (CI) algorithms. CI = 1, < 1 and > 1 indicated an additive effect, synergism and antagonism, respectively.

### Radiation of patient-derived glioblastoma cells

To establish the radiation-sensitisation effects of **1** on patient-derived glioblastoma cells, cells were pre-treated with 100 nM of each compound for 1 or 24 h, before cells were radiated with a single dose (3 Gy) of radiation. Following radiation, glioblastoma cells were fixed after 1 or 72 h. Cells were fixed after 1 h to investigate early radiation-induced alterations in DNA repair and double-stranded breaks, through the ratio of RAD51 to γH2AX foci, respectively. To investigate the radiation-sensitisation effects on the toxicity of **1**, cells were fixed after 72 h. Prior to fixation, 50 µL of media was removed to perform an LDH Assay and AlamarBlue (1:10) was added to the cells for 4 h and imaged prior to fixation. EdU (2.5 µM) was added 24 h prior to fixation, and cells were stained for apoptotic marker, cleaved-caspase 3 (CC3) using immunocytochemistry.

### Immunocytochemistry and image acquisition

Immunocytochemistry was performed following fixation. Primary antibodies used for experiments are described in Table 2. Images were acquired using the ImageXpress micro XLS™ (Molecular Devices) high-content screening system, housed at the Biomedical Imaging Research Unit, University of Auckland. Images were acquired from micro-well plates using the ×10/0.3 Plan or ×20/0.45 NA CFI Super Plan Fluor ELWD ADM objective lens.

**Table 2:** List of antibodies and dilutions used for immunocytochemistry and flow cytometry experiments

Antigen	Species	Company	Catalogue	ICC	Flow
**β-actin**	Rabbit	Abcam	Ab6276	1:5000	
**CC3**	Rabbit	Abcam	9661 L	1:1000	
**E2F1**	Mouse	Thermofisher	MA5-31589	1:500	
**γH2AX**	Mouse	Abcam	Ab26350	1:500	
**NFκB**	Rabbit	Santa Cruz	SC372	1:500	
**pRb**	Rabbit	Abcam	A184796	1:100	
**p53**	Mouse	Abcam	Ab26	1:100	
**RAD51**	Rabbit	Abcam	Ab63801	1:500	
**PE-TNFR1**	Mouse IgG2a	Biolegend	369,904		1:40
**TNFR1**	Mouse IgG2a	Biolegend	369,902		1:40

### Cytometric bead array

Cytometric bead array (CBA) was used to measure the levels of TNFα secreted into conditioned media using the Human TNF Enhanced Sensitivity Flex Set (BD Biosciences). Samples were diluted 1:5 and assayed according to the manufacturer’s instructions.

### siRNA knockdown of TNFR1

Glioblastoma cells were grown to confluence for 48 h before transfection. No target siRNA (siNT) or TNFR1 siRNA (siTNFR1) were diluted in DMEM:F12 with no additives, containing 3% Lipofectamine^®^ 3000 RNAiMAX (Thermofisher), and incubated at room temperature for 20 min. The siRNA:Lipofectamine^®^ mixture was added to plated cells at a 1:2 dilution of a 2X stock to give a final concentration of 50 nM siRNA and 0.3% Lipofectamine^®^. Glioblastoma cells were incubated with transfection mix for 24 h, before the second transfection of siTNFR1 and siNT for a further 24 h, with experiments begun on cells 24 h post-transfection when maximal knockdown was achieved.

### Flow cytometry analysis of TNFR1 cell surface expression

Flow cytometry was used to measure the cell-surface expression of TNFR1 on patient-derived glioblastoma cells, following treatment with **1** (100 nM).

#### Method A

Where internalisation was measured, cells were incubated with mouse monoclonal anti-TNFR1 for 15 min at room temperature. Cells were then briefly washed with assay medium and incubated with vehicle (DMSO, maximum concentration used was 0.1%) or **1** for the indicated period of time at 37 °C. A time course over 48 h was carried out in response to 100 nM of **1**, a concentration that induced a pronounced degree of anterograde trafficking at 24 h, to further investigate the time dependence of this trafficking phenotype. The application of primary antibody was staggered when Method A was used, so that the extent of constitutive internalisation could be measured over the time course. At the conclusion of drug stimulation, plates were placed on ice for a minimum of 2 min to halt membrane trafficking and briefly washed with room temperature assay medium. Alexa Flour^®^ 488-conjugated goat anti-mouse secondary (diluted 1:300 in assay medium) was then applied to the cells and incubated for 30 min at 15 °C to prevent further membrane trafficking. Because the monoclonal anti-TNFR1 antibody does not penetrate the cells, this method only detects TNFR1 receptors expressed on the cell-surface. Newly synthesized receptors cycling to the cell-surface are not detected. In contrast, Method B below measures all cell-surface TNFR1 including re-cycled or re-synthesized receptors.

#### Method B

Where net surface expression was measured, cells were incubated with shared vehicle or **1** for the indicated period of time at 37 °C prior to any labelling. At the conclusion of the drug stimulation, plates were placed on ice and washed as above. Following this, cells were incubated with mouse monoclonal anti-TNFR1 in assay medium for 15 min at 15 °C and then washed twice with assay medium. For both methods, cells were dissociated with 250 µL of Accutase for 4 min, before being equilibrated with assay medium and processed as described above.

#### Method C

Where total receptor expression was of interest, drug stimulations were carried out as described above, but cells were fixed in 500 µL 8% PFA for 10 min prior to centrifugation. Cells were then washed twice with 3 mL of PBS-T, and spun following each wash at 300 x g. Cells were then incubated with mouse monoclonal anti-TNFR1 diluted 1:100 in FACS buffer for 3 h at room temperature and washed with PBS-T. Alexa Flour^®^ 488-conjugated goat anti-mouse secondary (diluted 1:300 in assay medium) was then applied to the cells and incubated for 30 min.

A reduction in surface expression in Method A is indicative of receptor internalisation, and Method B measures net surface expression, which is a product of both internalisation and delivery of new receptors to the cell surface. The difference between these methods, therefore, reflects stimulated surface delivery of TNFR1.

### qRT-PCR analysis of gene expression

qRT-PCR was used to determine the mRNA expression of a panel of apoptosis and survival genes following treatment with palbociclib and **1**. Patient-derived glioblastoma cells were treated with 500 nM **1** (EC_50_), 10 µM palbociclib (EC_50_) and vehicle (0.01% DMSO) for 24 h. RNA was extracted from treated cells after 24 h using the RQ1 RNase-free DNAse kit (Promega, WI, USA) according to the manufacturer’s instructions. qRT-PCR was performed using Platinum® SYBR® Green qPCR SuperMix-UDG with Rox (Life Technologies) on a 7900HT Fast Real-Time PCR system (Applied Biosystems, CA, USA) for the genes and corresponding primers listed in Table 3. Standard curves were run for all primers and efficiencies were all 100 ± 10% (data not shown). Relative gene expression analysis was performed using the 2^-ΔΔCt^ method to the housekeeping gene B-actin.

**Table 3:** List of primers used for qRT-PCR

Accession	Gene (protein)	Sequence (5’ to 3’)		Amplicon (bp)
NM_001160.3	*APAF1*	Fw	CCAGTTCACAGCCGATGAGA	113
	(APAF1)	Rv	CTGTTTCCTGATGGCCTCGT	
NM_001101	*B-actin*	Fw	TGGTGGGCATGGGTCAGAAGGA	94
	(Β-actin)	Rv	ATGCCGTGCTCGATGGGGTACT	
NM_032989.3	*BAD*	Fw	CGGAGGATGAGTGACGAGTT	135
	(BAD)	Rv	CAAGTTCCGATCCCACCAGG	
NM_004324.3	*BAX*	Fw	GCCCTTTTCTACTTTGCCAGC	94
	(BAX)	Rv	AGTCCAATGTCCAGCCCATG	
NM_138578.3	*BCL2L1*	Fw	GAATCTCTTTCTCTCCCTTCAGA	117
	(Bcl-xL)	Rv	GCTCAACCAGTCCATTGTCC	
NM_000657.2	*Bcl-2*	Fw	ACTGGGGGAGGATTGTGGCCTT	70
	(BCL2)	Rv	ATCTCCCGGTTGACGCTCTCCA	
NM_004049.4	*BCL2A1*	Fw	AAATTGCCCCGGATGTGGAT	115
	(BFLM)	Rv	ACAAAGCCATTTTCCCAGCCT	
NM_138621.5	*BCL2L11*	Fw	TGATTCTTGCAGCCACCCT	116
	(BIM)	Rv	GGGGAACAAGGGCCAAGAAA	
NM_004346.3	*CASP3*	Fw	GGTGCTATTGTGAGGCGGTT	74
	(Caspase 3)	Rv	CCACGGATACACAGCCACAG	
NM_001080124.1	*CASP8*	Fw	TGGTCACTTGAACCTTGGGA	119
	(Caspase 8)	Rv	AGGGAGGCCAGATCTTCACT	
NM_003824.4	*FADD*	Fw	GCCCCTGTGTGAGTTGAGTC	72
	(FADD)	Rv	TCAATTCGTCCTGGCAACCA	
NM_001199649.2	*PTK2*	Fw	AGAAGAAAAGAATTGGGCGGA	135
	(FAK)	Rv	GGCTTGACACCCTCGTTGTA	
NM_000043.6	*FAS*	Fw	TGAACACTGTGACCCTTGCA	117
	(FAS)	Rv	AGACAAAGCCACCCCAAGTT	
NM_003806.4	*HRK*	Fw	GGAGCGAGCAACAGGTT	131
	(HRK)	Rv	CGCTGTCTTTACTCTCCACTTC	
NM_021960.5	*MCL1*	Fw	GGACAAAACGGGACTGGCTA	140
	(Mcl-1)	Rv	TGCCAAACCAGCTCCTACTC	
NM_182763.3	*MCL1*	Fw	TTGGCCTCAAAAGAAACGCG	120
	(Mcl-1)	Rv	CCTCCTTCTCCGTAGCCAAA	
NM_001012270.2	*BIRC5*	Fw	CCACTGAGAACGAGCCAGAC	89
	(Survivin)	Rv	CCTTTGCATGGGGTCGTCAT	
NM_001056	*TNFR1*	Fw	CTGGAGCTGTTGGTGGGAATA	76
	(TNFR1)	Rv	CTCTCTTCTCCCTGTCCCCT	
NM_000546.6	*TP53*	Fw	CTGGCCCCTGTCATCTTCTG	131
	(TP53)	Rv	ACATCTTGTTGAGGGCAGGG	
NM_01190942	*TNFSF10*	Fw	CACGACCAGGAACACAGCAT	139
	(TRAIL)	Rv	GATGTCACTCCAGGGCGTAC	

### Proteome profiler; human NFkB array

Patient-derived glioblastoma cells were cultured and harvested as previously described [42]. Patient-derived glioblastoma cells were pre-treated with vehicle (0.01% DMSO) or 500 nM **1** for 24 h, followed by a brief wash with assay medium, and treatment with 50 µg/mL TNFα or vehicle (0.1% BSA) for a further 15 min. Cell lysis solutions were assayed using the Proteome Profiler™ Human NFκB Array Kit as per the manufacturer’s instructions (R&D Systems, MN, USA).

### Statistical analysis

All sigmoidal concentration-response curves were obtained by fitting three-parameter (Hill slope constrained to 1) nonlinear regression curves (GraphPad Prism, v8; GraphPad Software Inc., La Jolla, CA) and all graphs were prepared in GraphPad Prism or RStudio. Statistical analyses were then performed on the means from at least three independent experiments using Graphpad and Sigmaplot™ v13.0 (Systat Software Inc., San Jose, CA, USA). The ShapiroWilk test for normality and Brown-Forsythe test for equal variance were performed to verify that the datasets were appropriate for analysis with parametric statistical tests. Where a set of results did not pass either of these tests, datasets were transformed (log10 or e^x^ ) to change the nominal values of the sample distribution, and re-tested. Following a pass result of *p* > 0.05, a Student’s t-test, one-way ANOVA, or two-way ANOVA was carried out as appropriate for the number of conditions and factors under comparison. A paired or repeated measures design was used where appropriate. If a statistically significant difference (*p* < 0.05) was detected in any of the above tests, the Holm-Šídák [43] post-hoc test was used to test for significant differences within/between groups.

## Results

### Conjugation of cyclin-dependent kinase inhibitor, palbociclib with MHI 148 significantly improves their potency in patient-derived glioblastoma cells

**Fig. 1:**
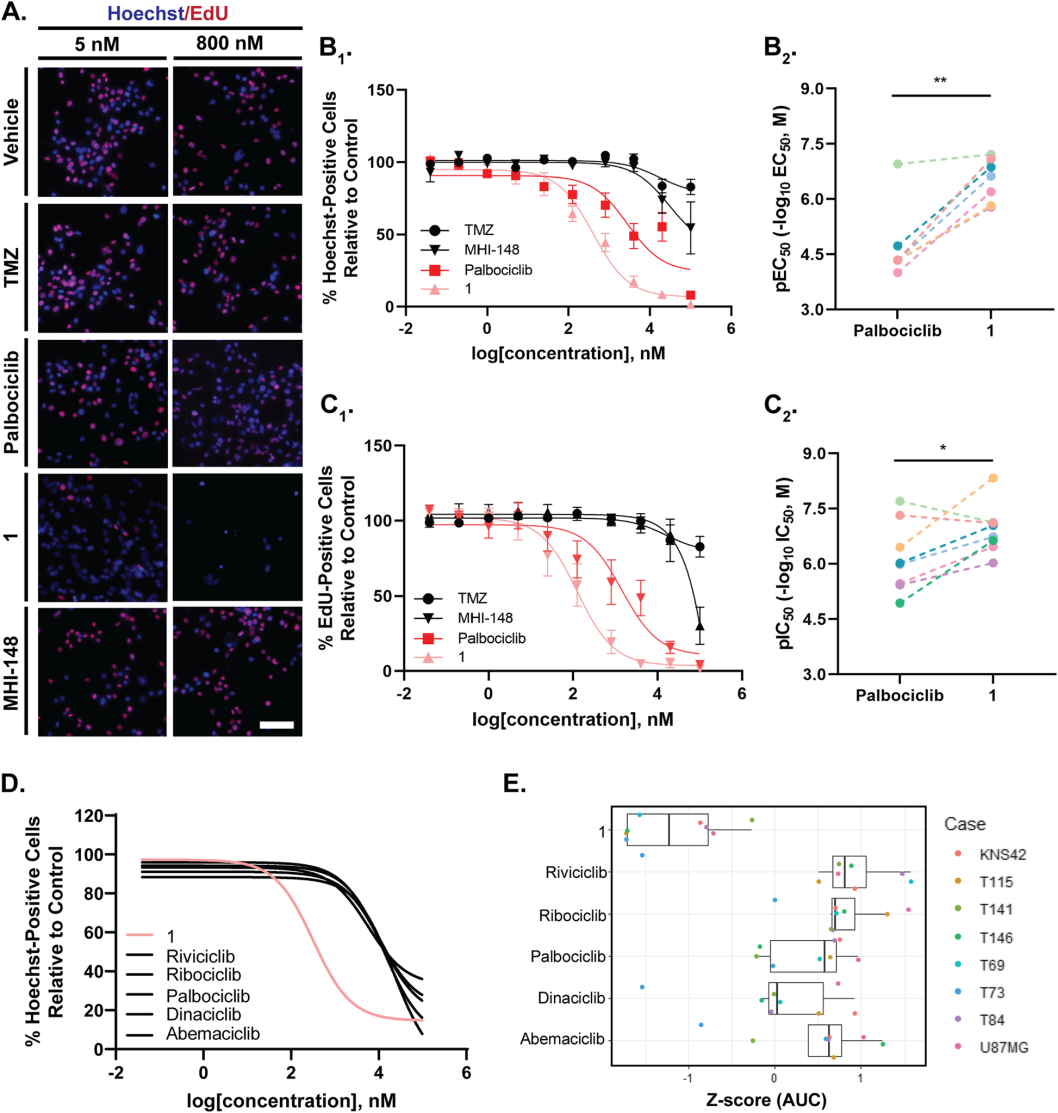
Viability and antiproliferative activity of cyclin-dependent kinase inhibitor palbociclib conjugated to MHI-148 [1] in patient-derived glioblastoma cells. Patient-derived glioblastoma cells were treated with DMSO (vehicle), and up to 100 µM of TMZ, palbociclib, **1** and MHI-148 for 48 or 96 h. Concentration-dependent effects of each compound on the toxicity of glioblastoma cells were measured by the percentage of Hoechst-positive cells after 96 h (EC_50_, B_1_) with cell-line specific pEC_50_ for **1** relative to palbociclib summarised in B_2_. Concentration-dependent effects of each compound on the proliferative activity of glioblastoma cells were measured using the percentage of EdU-positive cells after 48 h (IC_50_) (C_1_) with cell-line specific pEC_50_ for **1** relative to palbociclib summarised in C_2_. **A** non-linear curve was fitted using GraphPad Prism using the concentration of each compound versus the total number of cells (**B**) or the percentage of EdU-positive cells normalised to DMSO (**C**). **1** was significantly more cytotoxic than palbociclib, as evident by the pEC50 activity (B1) and **1** is more potent at inhibiting the percentage of EdU-positive cells (C1). The Potency of **1** was evaluated against five other CDK inhibitors (**D**, **E**). Distribution of Z-scores for each drug’s AUC (**E**). Each dot represents an individual glioblastoma case. Data represents mean ± SEM * = *p* < 0.05 ** = *P* < 0.0001 to Palbociclib (B1, C1), paired students t-test. Data displayed is from at least six independent glioblastoma cases

The CDK4/6 inhibitor palbociclib-MHI-148 conjugate, **1**, was screened against six different patient-derived glioblastoma cell lines, obtained from Auckland City Hospital (Table 1). Evidently, the palbociclib-MHI-148 conjugate [1] was significantly more potent at inhibiting the proliferation of glioblastoma cells relative to palbociclib treatment (IC_50_: 111 ± 83.7 nM, 2316 ± 4501 nM, respectively, *p* = 0.0391, Fig. 1C and Table 4). Interestingly, whilst palbociclib had minimal cytotoxic activity on glioblastoma cells, conjugation of palbociclib with MHI-148 resulted in significant cytotoxic activity; **1** significantly reduced the total number of Hoechst-positive nuclei relative to palbociclib (EC_50_: 33 ± 19 µM, 369 ± 285 nM, *p* = 0.0078, respectively, Fig. 1B). Given the potent cytotoxic activity of **1**, we compared the potency of **1** with five FDA-approved CDK inhibitors including CDK4/6 inhibitors, palbociclib, ribociclib, and abemaciclib and CDK9 inhibitors, riviciclib and dinaciclib (Fig. 1D, E). Indeed, **1** was considerably more potent at reducing the percentage of Hoechst-positive cells, as indicated by the left shift in the concentration response (Fig. 1D) and the left-shift in the median z-score (Fig. 1E) for **1** in comparison to the other CDK inhibitors.

**Table 4:** IC _50_, pIC _50_, EC _50_ and pEC _50_ of the proliferation and viability of glioblastoma cells using the EdU cell proliferation assay (48 h) and Hoechst as a marker of total cell count (96 h), respectively, normalised to DMSO.

		Palbociclib		1	
	**Combination**	**-TMZ**	**+TMZ**	**- TMZ**	**+ TMZ**
**Edu**	**IC**_**50**_ **(nM) [±SEM]**	2316 [4501]	171 [180]	111 [83.7]	66.3 [68.5]
**(Proliferation, 48h)**	**pIC**_**50**_ **(log**_**10**_ **, M) [±SEM]**	6.4 [1.0]	7.1 [0.7]	7.2 [0.6]^*^	7.7 [0.9]
**Total Cell Number**	**EC**_**50**_ **(nM) [±SEM]**	33260 [19400]	41540 [48600]	352 [575]	415 [164]
**(Cytotoxicity, 96h)**	**pEC**_**50**_ **(log**_**10**_ **, M) [±SEM]**	4.8 [1.0]	4.9 [0.9]	6.6 [0.5]^**^	6.4 [0.18]

The IC_50_ (nM) and the pIC_50_ (-log_10_, IC_50_, M) were determined using EdU as an index of the percentage of proliferating cells. The EC_50_ and the pEC_50_ (-log_10_, EC_50_, M) was determined using Hoechst as an index of the total number of cells. A non-linear curve was fitted using GraphPad Prism using the concentration of each compound versus the relevant measure, normalised to vehicle (DMSO). Data displayed is from six independent glioblastoma cases. Data represents mean ± SEM. **** = *P* < 0.0001, *** = *P* < 0.001, ** = *P* < 0.01, * = *P* < 0.05, ns = *P* > 0.05, of the pIC_50_ or pEC_50 _of **1 ** to palbociclib (One-way ANOVA with Tukey’s multiple comparisons test)

### Palbociclib-MHI 148 conjugate, 1, is a potent radiosensitizer in patient-derived glioblastoma cells

**Fig. 2:**
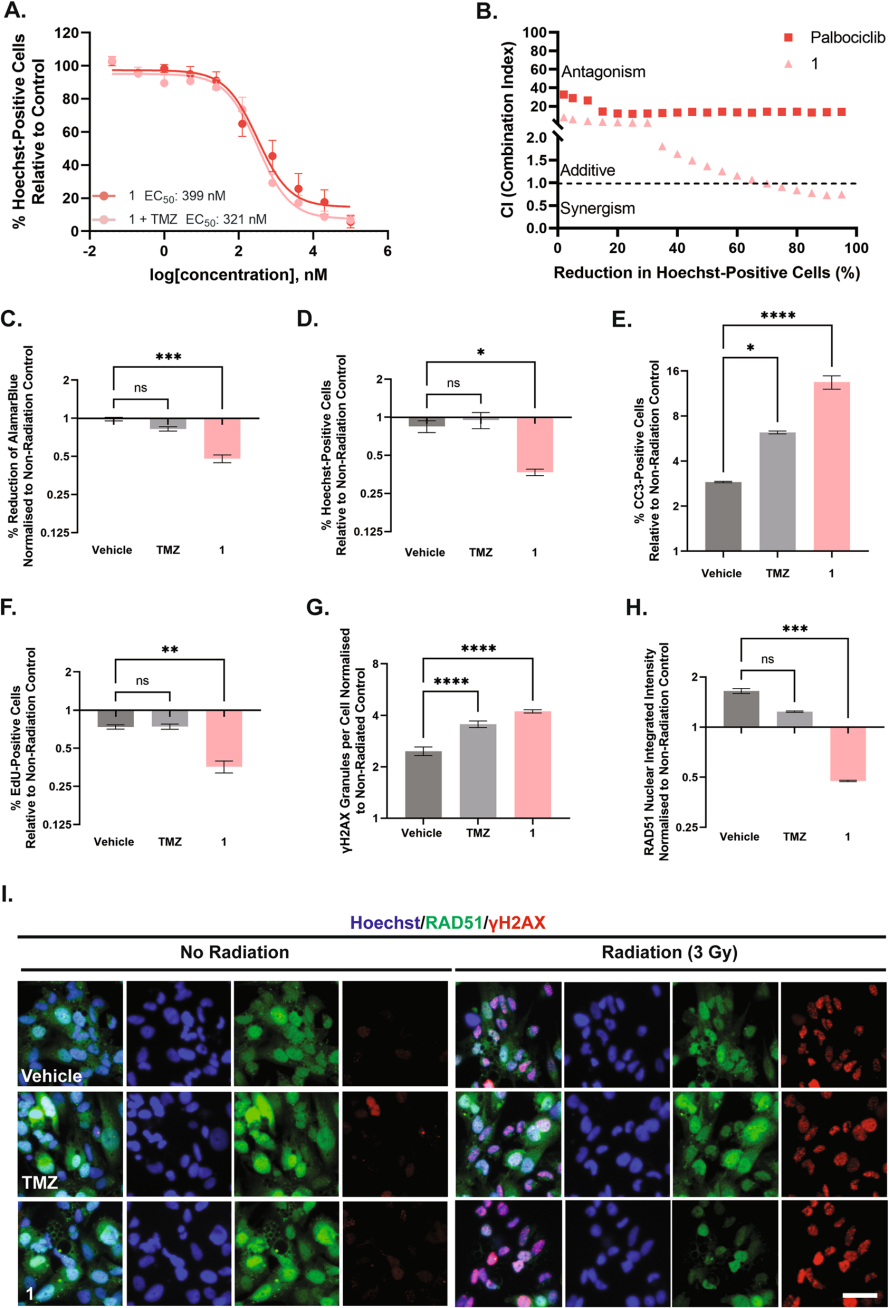
Palbociclib-MHI-148 conjugate, 1 is not synergistic with TMZ, but is synergistic with a single dose (3 Gy) of radiation. Patient-derived glioblastoma cells were treated with up to 100 µM of palbociclib, **1**, with and without 100 µM of TMZ for 96 h. Concentration-dependent effects of each compound on the toxicity of glioblastoma cells were measured by the percentage of Hoechst-positive cells after 96 h (EC_50_) (**A**). A non-linear curve was fitted using GraphPad Prism using the concentration of each compound versus the percentage of Hoechst-positive cells (A). Combination index (CI) was calculated from the CI equation algorithms using CompuSyn software. CI = 1, < 1 and > 1 indicates additive effect, synergism, and antagonism, respectively (**B**). The effects of **1**, with and without radiation, were evaluated in terms of the viability (**C**) as indicated by the reduction in alamarBlue and percentage of Hoechst-positive nuclei (**D**), in addition to the activation of apoptosis marker cleaved-caspase 3 (**E**) and proliferation marker, EdU (**F**). The radiation sensitisation effects of **1** on the induction of double-stranded breaks were quantified using the number of γH2AX granules per cell (**G**) and the mean nuclear integrated intensity of RAD51 (**H**). Representative immunocytochemical staining of γH2AX and RAD51 in T146 is seen in I. Data represents mean ± SEM for four independent glioblastoma cases, ns = *p* > 0.05, * = *p* < 0.05, ** = *p* < 0.01, *** = *p* < 0.001, *** = *p* < 0.0001, relative to vehicle

Given the significant increase in cytostatic potency of **1** relative to palbociclib, in addition to its potent cytotoxic activity, we sought to evaluate the potency in combination with the standard of care treatments of glioblastoma, TMZ and radiation (Fig. 2). Notably, when co-incubated with 100 µM TMZ, palbociclib was 14-fold (IC_50_: 171 ± 180 nM, versus 2316 ± 4501 nM) more potent at inhibiting proliferation compared to palbociclib treatment alone (Table 4). However, co-incubation of **1** with TMZ only increased the potency by 1.6-fold (111 ± 83.7 nM, versus 66.3 ± 68.5 nM) relative to **1** treatment alone (Table 4). Whilst the combination of **1** with TMZ saw considerable improvement in the anti-proliferative effects (48 h), this was not reflected in the cytotoxicity of **1** (96 h), as indicated by their respective pEC_50_ values (Table 4). The lack of consistency was reflected in the variable response to co-incubation with TMZ when assessed on a case-by-case basis (Supplementary Fig. 1). However, using the CompuSyn algorithm, when combining the combination indices of each case, co-incubation of palbociclib with TMZ, in fact, antagonised the reduction in the percentage of Hoechst-positive cells (Fig. 2B). In contrast, combining **1** with equivalent concentrations of TMZ trended towards a synergistic reduction in the percentage of Hoechst-positive cells, particularly at higher effect sizes (Fig. 2B). However, when comparing the combination indices of each compound within each case, the outcome is less clear (Supplementary Fig. 1). Co-incubation of palbociclib with TMZ was synergistic at higher effect levels (> 25% reduction in Hoechst-positive cells), and this was maintained in **1** in all cases except U87MG, which results in antagonism, and T73, which was additive (Supplementary Fig. 1). This synergistic response did not correlate with the MGMT methylation status of these cell lines summarised in Table 1.

Despite the lack of synergism with TMZ across GBM cell lines, we further sought to evaluate the synergistic effect of **1** with a single dose (3 Gy) of radiation (Fig. 2). Interestingly, we found that pre-treatment of glioblastoma cells with **1** resulted in significantly greater cytotoxic activity, as indicated by a fold change in the percentage reduction in alamarBlue™ (Fig. 2C, *p* = 0.0008) and Hoechst-positive nuclei (Fig. 2D, *p* = 0.0259), in addition to the significant increase in the percentage of CC3-positive nuclei (Fig. 2E, *p* < 0.0001). Moreover, pre-treatment with **1** (Fig. 2F, *p* = 0.0057) significantly reduced the percentage of EdU-positive nuclei. Notably, pre-treatment with **1** was more effective than pre-treatment with TMZ, an established radiosensitizer, across all the modalities described (Fig. 2C-I). This was further reinforced by a significant increase in the number of γH2AX-foci within the nucleus of glioblastoma cells, an indicator of DNA double-stranded breaks (Fig. 2G, *p* < 0.0001) and a concurrent reduction in the mean nuclear intensity of DNA-repair marker, RAD51 (Fig. 2H, *p* = 0.0002). Overall, these data suggested that despite the lack of synergism with TMZ, **1** was a potent radiosensitizer when administered alongside a single dose of radiation.

### The cytotoxic activity of palbociclib-MHI 148 conjugate is driven by a TNFR1-dependent cell death pathway

Evidently, conjugation of CDK inhibitor, palbociclib, with MHI-148 resulted in a potent cytotoxic compound, **1**. The shift in the modality of palbociclib from cytostatic to cytotoxic when conjugated with MHI-148 was intriguing; hence, we sought to investigate the mechanisms of action of **1**.

**Fig. 3:**
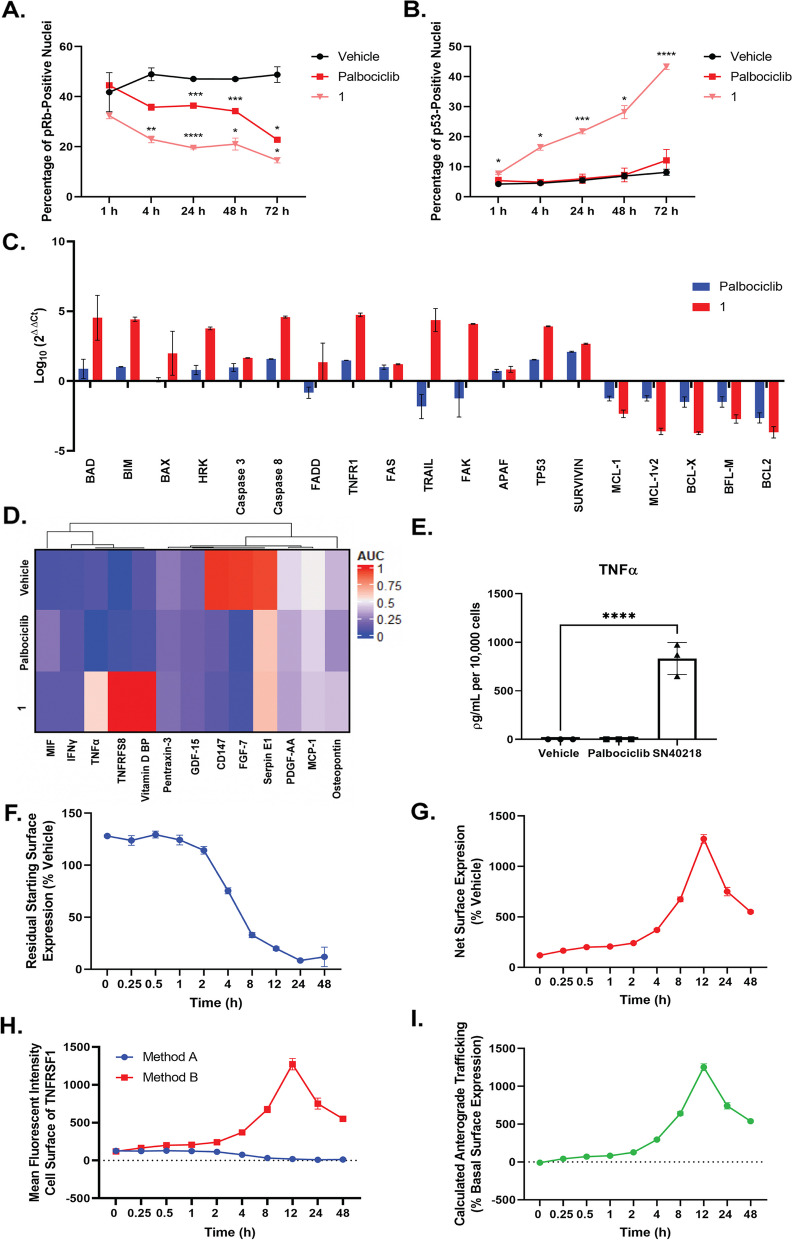
Treatment of glioblastoma cells with palbociclib-HMCD conjugate increases the expression of soluble TNFα, TNFRSF8 and cell surface expression of TNFR1 prior to the induction of cell death. pRb (**A**) and p53 (**B**) expression in response to vehicle (0.01% DMSO), palbociclib (100 nM) or 1 (100 nM) for up to 72 h. Glioblastoma cells were treated with palbociclib (100 µM) and 1 (200 nM) for 24 h to investigate transcription of cell death and pro-survival genes (**C**). Following treatment with palbociclib (100 µM), 1 (200 nM) or vehicle (0.01% DMSO), conditioned media was extracted for proteome profiler (**D**) and CBA (**E**) analysis. Residual starting surface expression TNFR1 (Method A) (**F**). To investigate the net surface expression of TNFR1 (Method B) (**G**). **H** summarises methods A and B detection of TNFR1. Calculated anterograde trafficking of TNFR1 (Method B-Method A) (**I**). Data represents mean ± SEM from three independent glioblastoma cases. *p* < 0.05, * *p < 0.01, *** *p* < 0.001, **** *p* < 0.0001 relative to vehicle at each respective time point (Two-way ANOVA)

Whilst we reported potent inhibition of glioblastoma cell proliferation with **1**, given the unique cytotoxic profile following **1** treatment, we sought to validate whether **1** was still inhibiting CDK4/6 (Fig. 3A). As aforementioned, the principal function of cyclin D-CDK4/6 activity is to phosphorylate pRb at sites that prevent it from binding to members of the E2F family of transcription factors, which control the expression of genes that support DNA synthesis and S-phase progression [44]. Hence, we investigated whether treatment with **1** inhibited pRb phosphorylation in glioblastoma cells. Indeed, we demonstrate a time-dependent inhibition of phosphorylated pRb from as early as 1 h in **1**-treated cells (Fig. 3A). Moreover, the extent of phosphorylated-pRb inhibition was substantially greater in **1**-treated cells compared to palbociclib-treated cells, at the same concentration (Fig. 3A). Consistent with our observation on the cytotoxicity of **1**, we observed a concurrent increase in p53-positive nuclei over the course of 72 h in **1**-treated cells but not palbociclib-treated cells (Fig. 3B).

Following treatment with the approximate cytotoxic EC_50_ of **1** (300 nM, 24 h), we observed a clear increase in the transcription of key intrinsic (*BAD, BIM, BAX, HRK*) and extrinsic (*Caspase 8, FADD, Fas, TRAIL, TNFR1*) apoptosis genes, and a reduction in the transcription of pro-survival genes (Fig. 3C). Of particular interest was the clear increase in the transcription of death receptors TNFR1 and TRAIL, and hence we sought to determine whether there was a concurrent increase in the secretion of the death receptor ligand, TNFα. A human cytokine XL array was used to screen the secretome of glioblastoma cells in response to palbociclib (100 µM) and **1** (200 nM) (Fig. 3D). Interestingly, treatment of glioblastoma cells with **1** resulted in significant TNFα secretion (*p* < 0.0001, one-way ANOVA), relative to both vehicle and palbociclib treatment (Fig. 3E).

Given TNFα was specifically secreted by glioblastoma cells treated with **1** but not palbociclib, we investigated the role of TNFα in the context of the death receptor TNFR1(Fig. 3F-I). We used different receptor measuring methods to determine the effects of **1** on cell-surface expression (internalisation) of TNFR1 (Method A) and the expression of TNFR1 from both existing cell-surface receptors and re-cycled/re-synthesized receptors (Methods B and C). A reduction in surface expression in Method A is indicative of receptor internalisation, and Method B measures net surface expression, which is a product of both internalisation and delivery of new receptors to the cell surface. The difference between these methods, therefore, reflects stimulated surface delivery of TNFR1.

A time course over 48 h was carried out in response to 200 nM **1** (EC_50_) to further investigate the time-dependence of this trafficking phenotype. The application of primary antibody was staggered when Method A was used, so that the extent of constitutive internalisation could be measured over the time course; however, constitutive internalisation of TNFR1 was not observed (data not shown). Approximately 95% of surface TNFR1 internalised in response to **1** between 24 and 48 h, with significant internalisation of the surface expression beginning from as early as 4 h (*p* < 0.0001) (Fig. 3F). When net surface expression was measured (Method B) in response to **1**, TNFR1 surface expression increased in a time-dependent manner to a maximum at 12 h, followed by a sharp decrease in net surface expression between 24 and 48 h (Fig. 3G). Subtraction of Method A (internalisation) from Method B (net surface expression), revealed the anterograde trafficking/surface delivery of TNFR1 that took place over the 48 h time course (Fig. 3H, I). Anterograde trafficking of TNFR1 in response to **1** was significantly different from vehicle at all time points > 1 h, (1 h: *p* = 0.0484, 2 h; *p* = 0.0022, 4–48 h; *p* < 0.0001, relative to 0 h) indicating that this phenotype occurs following a lag time of at least 1 h (Fig. 3I). Surface delivery of TNFR1 in response to **1** continued at a steady rate up to 12 h, followed by internalisation from 24 h (Fig. 3I).

### The induction of TNFR1-dependent cell death by 1 required endocytosis of TNFR1 and synthesis and delivery of new TNFR1 to the plasma membrane

**Table 5:** Trafficking summary data for TNFR1 in response to 1, in the absence or presence of vesicle trafficking inhibitor, BFA, and protein synthesis inhibitor, CHX. E_max_ expressed as a percentage of vehicle. Parameters are derived from at least three independent patient-derived GBM cases. ns = *p* > 0.05, ** *p* < 0.01, *** p < 0.001 relative to 1 with vehicle, One-way ANOVA

**1-induced anterograde trafficking of TNFR1 (12 h)**			
	**+ Vehicle**	**+ BFA**	**+ CHX**
**EC**_**50**_ **(nM) [±SEM]**	63.3 [1.5]	481.3 [12.9]	779.8 [293.2]
**pEC**_**50**_ **(log**_**10**_ **, M) [±SEM]**	7.20 [0.01]	6.32 [0.01]***	6.14 [0.17]**
**E**_**max**_ **(nM)[±SEM]**	4184 [2348]	3835 [2330]^ns^	3559 [1975]^ns^
**1-induced total TNFR1 expression (12 h)**			
**EC**_**50**_ **(nM) [±SEM]**	44421.7 [30.05]	122.9 [23.09]	1154 [15.6]
**pEC**_**50**_ **(log**_**10**_ **, M) [±SEM]**	6.38 [0.03]	6.92 [0.08]*	5.94 [0.01]*
**E**_**max**_ **(nM)[±SEM]**	6625 [265]	8994 [203]**	5943 [307]**

**Fig. 4:**
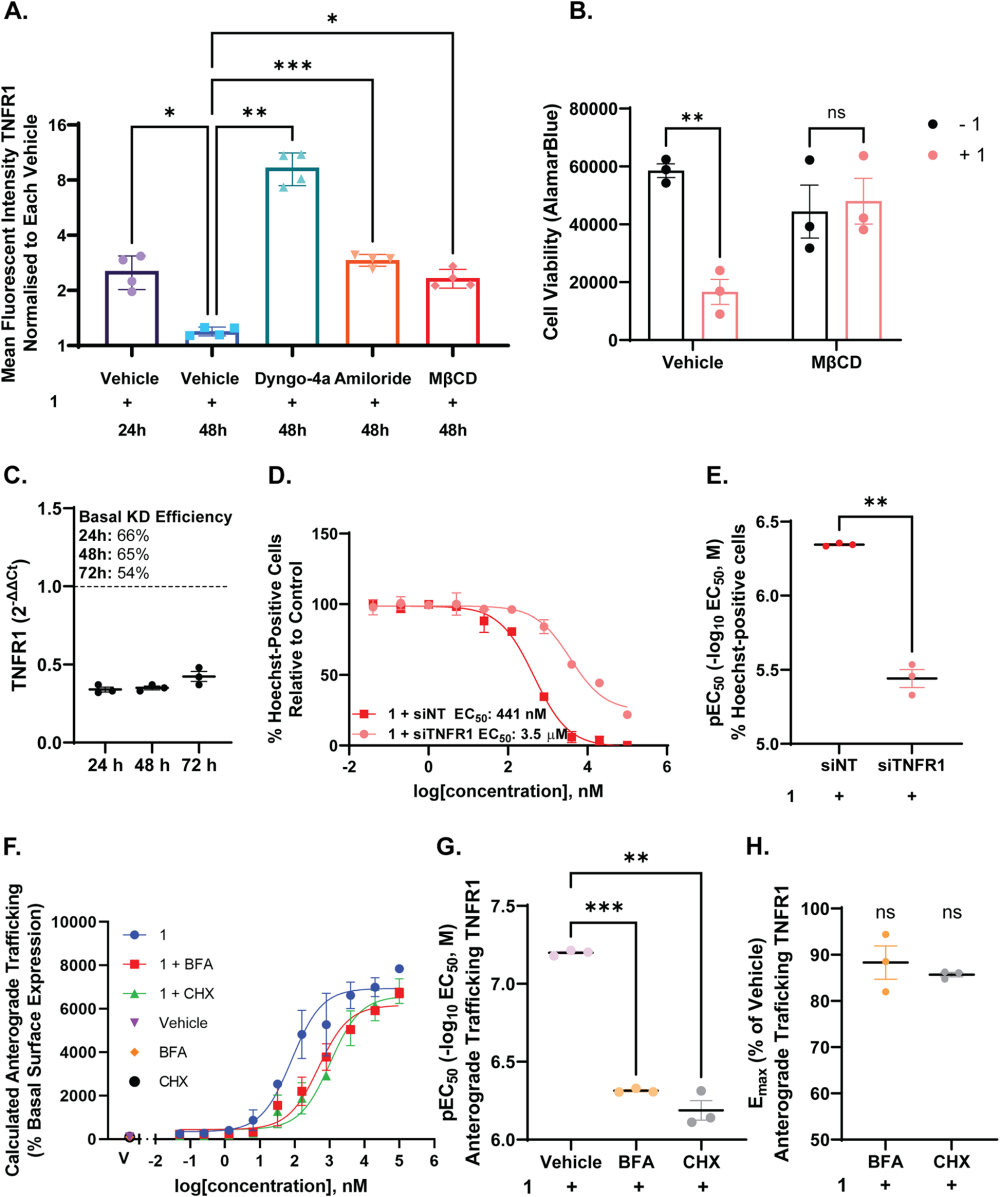
TNFR1 endocytosis is required for 1-induced cell death. Calculated anterograde trafficking of TNFR1 in response to **1** ± BFA or CHX (Method B-Method A) (**A**). pEC50 and Emax reported in **B** and **C**, respectively. The mean fluorescent intensity of TNFR1 cell surface expression (Method B) after 48 h was investigated in the presence or absence of pre-treatment with endocytosis inhibitors, dyngo-4a (30 µM), amiloride (100 µM), or MβCD (100 µM) in the presence of **1** (100 nM) (**D**). Inhibition of endocytosis with MβCD (100 µM), prevents **1**-induced cell death, as indicated by alamarBlue™ (**E**). siRNA knockdown of TNFR1 reduced the potency of **1** in patient-derived glioblastoma cells (**F**-**H**). Data represents mean ± SEM from at least three independent glioblastoma cases. ** *p* < 0.01, *** *p* < 0.001 relative to **1** with inhibitor vehicle

To investigate the anterograde trafficking of TNFR1 in response to **1**, we investigated whether inhibitors of receptor trafficking and protein synthesis altered the **1**-induced TNFR1 trafficking phenotype using the same labelling methods described above (Fig. 4F). Brefeldin A (BFA) is a macrocyclic lactone that disrupts the Golgi and prevents protein secretion to the plasma membrane, and this would be expected to inhibit the cell surface delivery of TNFR1 [45]. The presence of CHX, a protein synthesis inhibitor, was included to determine whether new protein synthesis was required for this phenotype to occur [46].

Treatment with BFA and CHX had no effect on the internalisation of residual TNFR1 in response to **1** (Method A) (Supplementary Fig. 2). However, treatment with BFA significantly reduced TNFR1 net surface expression in response to **1**, producing a concentration-response curve in this labelling condition with a moderate reduction in efficacy (12% [± 3.6]) and potency (481.3 [± 21.9] nM vs. 63.3 [± 1.5] nM, *p* = 0.0003) to that when measuring net surface expression of TNFR1 in response to **1** alone (Table 5; Fig. 4F-H). **1**-induced anterograde trafficking was therefore moderately reduced when BFA was present (Fig. 4, F). Furthermore, inhibiting protein synthesis with CHX treatment also resulted in a decrease in net surface expression of TNFR1 following **1** stimulation, and, therefore, partial blockade of the stimulated anterograde trafficking phenotype, reducing its potency (779.8 [± 293.2] nM, *p* = 0.0050) (Table 5; Fig. 4F-H). Interestingly, BFA treatment resulted in a significant increase in total TNFR1 expression at high concentrations of **1**, suggesting **1** had an effect on TNFR1 expression even when the delivery of TNFR1 was inhibited (EC_50_ 122.9 [23.09] nM, E_max_ 136% relative to **1** alone) (Table 5, Supplementary Fig. 2). CHX treatment significantly reduced the total **1**-induced expression of TNFR1 (EC_50_ 1154 [15.6] nM, E_max_ 86% relative to **1** alone ), which was unsurprising given intracellular TNFR1 would have to use the secretory pathway to be delivered to the cell surface (Table 5, Supplementary Fig. 2). However, it is important to note that CHX is non-specific and blocks all protein synthesis. Overall, these data suggested that the TNFR1 anterograde trafficking phenotype relied on a combination of new protein synthesis and transport of newly synthesised protein to the plasma membrane. Furthermore, this supports the notion that **1** may have a chaperone effect on TNFR1, stabilising new receptors and preventing cleavage and degradation of TNFR1.

TNFR1-endocytosis through clathrin-coated vesicles, following TNFα ligand binding, is required for TNFR1-apoptosis signalling [47–49]. In contrast, TNFα-induced canonical TNFR1 signalling occurs from the cell surface [47–49]. To validate that the reduction in **1**-induced cell surface TNFR1 expression was indeed due to endocytosis, and not cleavage, we investigated the effects of endocytosis inhibitors on TNFR1 expression (Fig. 4A, B). Indeed, treatment with dynamin-inhibitor dyngo-4a (*p* = 0.0087), macropinocytosis inhibitor amiloride (*p* = 0.0001), and lipid-raft inhibitor MβCD (*p* = 0.0007), prevented **1**-induced endocytosis of TNFR1 at 48 h (Fig. 4A). This was indicated by the significant increase in the mean fluorescent intensity of cell surface TNFR1 expression, relative to **1**-treated cells (Fig. 4A, *p* < 0.0001). Interestingly, inhibition of endocytosis with dyngo-4a and amiloride increased the mean fluorescent intensity of TNFR1 further than the initial 24-h peak, this was likely a consequence of stabilisation of TNFR1 at the plasma membrane, thereby preventing constitutive TNFR1 endocytosis (Fig. 4A). Furthermore, pre-treatment of glioblastoma cells with endocytosis inhibitor MβCD (*p* = 0.9935, Sidaks multiple comparisons test) prevented **1**-induced cell death (Fig. 4B). To further validate the role of TNFR1 in **1**-induced cell death, TNFR1 knockdown with siTNFR1 resulted in a significant right shift in the potency of **1**-dependent cell death after 72 h (siNT: 441 nM, siTNFR1: 3.5 µM, *p =* 0.0048*)*, reinforcing that TNFR1 was required for 1-induced cell death (Fig. 4C-E).

### 1 as a sensitizer to TNFα/TNFR1-dependent apoptosis through nuclear factor (NF)-κB suppression

**Fig. 5:**
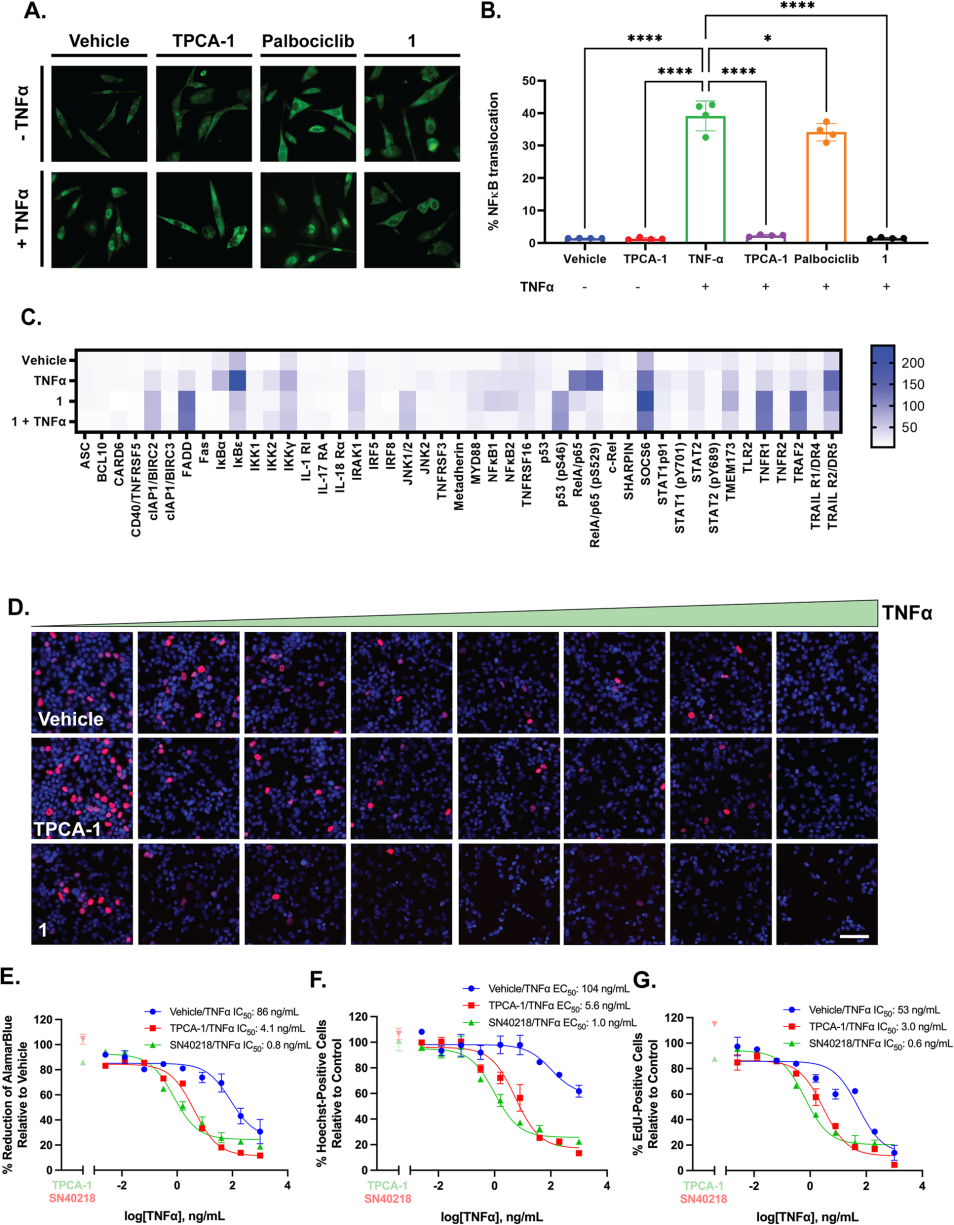
1 prevents TNFα-induced NFκB translocation, favouring non-canonical TNFR1 death receptor signalling in patient-derived glioblastoma cells and sensitizes glioblastoma cells to TNFα treatment (**A**). Glioblastoma cells were pre-treated with palbociclib (100 µM, 24 h), 1 (100 nM, 24 h), TPCA-1 (10 µM, 1 h) or vehicle (0.1% DMSO, 24 h), followed by treatment with TNFα (50 µg/mL) or vehicle (0.1% BSA) for 15 min (**A**, **B**). The percentage of nuclear NFκB-positive cells was used to determine the percentage of NFκB translocation (**B**). Representative images are seen in A. Human NFκB Proteome Profiler was used to investigate early TNFR1-signalling pathways following pre-treatment with 1 (100 nM, 24 h) and TNFα (50 µg/mL, 15 min), presented as a heatmap of the AUC (**C**). IC_50_, pIC_50_, EC_50_ and pEC_50_ of the viability (**E**), toxicity (**F**) and proliferative activity (**G**) of glioblastoma cells normalised to the vehicle (0.1% BSA/PBS, 0.01% DMSO). The IC_50_ (nM) and the pIC_50_ (-log_10_, IC_50_, M) were determined using EdU as an index of the percentage of proliferating cells, and the percentage reduction of alamarBlue fluorescent intensity as an index of cell viability. The EC_50_ and the pEC_50_ (-log_10_, EC_50_, M) were determined using Hoechst as an index of the total number of cells. A non-linear curve was fitted using Graphpad Prism using the concentration of TNFα versus the relevant measure, normalised to the vehicle (0.1% BSA/PBS, 0.01% DMSO) (three parameters). Data represents mean ± SEM from at least four independent glioblastoma cases, * *p* < 0.05, *** *p* < 0.0001

As aforementioned, TNFR1 signalling occurs through two sequential signalling complexes, a membrane-bound complex (complex 1) consisting of TNFR1, TRADD, RIP1 and TRAF2, and a cytosolic complex (complex 2) in which TRADD and RIP1 associate with FADD and caspase 8 [48]. Canonical pro-survival signalling relies on the activation of NFκB by complex 1, leading to the recruitment of FLIP, an inhibitor of caspase-8 activation, to complex 2. In contrast, in the absence of NFκB translocation, cells undergo apoptosis via complex 2 [48]. Therefore, we hypothesised that treatment with palbociclib (100 µM, 24 h)and **1** would prevent TNFα-induced NFκB translocation. We stimulated TNFα-induced (50 µg/mL, 1 h) canonical NFκB signalling with and without pre-treatment with **1** (100 nM, 24 h), NFκB inhibitor, TPCA-1 (10 µM, 1 h) or vehicle (0.01% DMSO) (Fig. 5). As expected, pre-treatment with TPCA-1 significantly inhibited TNFα-induced NFκB translocation (*p* < 0.0001, Fig. 5). Conforming to our hypothesis, pre-treatment with palbociclib (*p* = 0.0181) and **1** (*p* < 0.0001) significantly inhibited TNFα-induced NFκB translocation (Fig. 5A, B). Whilst palbociclib (100 µM) also significantly inhibited TNFα-induced NFκB translocation, it only reduced the percentage of NFκB translocation by approximately 10% (Figue 5 B). In contrast, pre-treatment with **1** completely inhibited NFκB translocation, and was achieved at a much lower concentration (100 nM) (Fig. 5B). Given that NFκB translocation is the final transduction step in NFκB signalling, we used a Human NFκB Proteome Profiler to investigate the earlier events in canonical and apoptotic TNFR1-signalling in response to **1** (Fig. 5C). Patient-derived glioblastoma cells were pre-treated with **1** (100 nM) or vehicle (0.01% DMSO) for 24 h, followed by TNFα (50 µg/mL, 15 min) or vehicle (0.1% BSA/PBS, 15 min). The array revealed that pre-treatment with **1** reduced TNFα-induced early canonical NFκB proteins (Fig. 5C). This included reductions in TNFα-induced IκBα/ε/γ, IKK1/2, NFκB1/2 and phosphorylation of RelA/p65 (pS529) (Fig. 5C). Concurrently, we saw an increase in non-canonical TNFR1-signalling including cIAP1/2, JNK, phosphorylation of p53 (pS46), TRAF2, and, as expected, TNFR1 in response to treatment with **1** (Fig. 5C).

Given the ability of **1** to induce TNFα-TNFR1-dependent cell death signalling, we hypothesised that treatment with sub-toxic concentrations of **1** (50 nM) would sensitize glioblastoma cells to treatment with TNFα. Moreover, inhibition of NFκB translocation has been shown to sensitize glioblastoma cells to TNFα treatment; hence, IKK inhibitor, TPCA-1 (10 µM) was used as a positive control [50, 51]. Indeed, we found that pre-treatment with **1** (50 nM) significantly increased the potency of TNFα (1.25 ± 0.47 ng/mL, *p* = 0.0139) relative to both TPCA-1 pre-treatment (4.13 ± 0.3 ng/mL) and TNFα alone (131.5 ± 19.3 ng/mL) (Table 6; Fig. 5). This trend was consistent across the read-outs summarised in Table 6. These results suggest that **1** sensitized glioblastoma cell to TNF-α-induced apoptosis, at least in part, via the suppression of NFκB.

**Table 6:** Summary data of the sensitization of patient-derived glioblastoma cells to TNFα

	Combination	Vehicle + TNFα	TPCA-1 + TNFα	1
**AlamarBlue (nM)**	**IC**_**50**_ **[±SEM]**	131.5 [19.3]	4.13 [0.30]^*^	1.25 [0.47]^*, ##^
**(Viability)**	**pIC**_**50**_ **[±SEM]**	6.89 [0.07]	8.39 [0.03]	9.0 [0.17]
**Total Cell Number (nM)**	**EC**_**50**_ **[±SEM]**	94.5 [12.5]	8.43 [3.9]^**^	1.25 [0.32]^**, ns^
**(Toxicity)**	**pEC**_**50**_ **[±SEM]**	7.04 [0.05]	8.3 [0.28]	8.95 [0.12]
**EdU (nM)**	**IC**_**50**_ **[±SEM]**	96.4 [16.1]	6.04 [3.4]^*^	0.98 [0.3]^*, ns^
**(Proliferation)**	**pIC**_**50**_ **[±SEM]**	7.04 [0.08]	8.39 [0.21]	9.07 [0.13]

IC_50_, pIC_50_, EC_50_ and pEC_50_ of the viability (B), toxicity (C) and proliferative activity (D) of glioblastoma cells normalised to vehicle (0.1% BSA/PBS, 0.01% DMSO).  The IC_50_ (ng/mL) and the pIC_50_ (-log10, IC_50_, M) were determined using EdU as an index of the percentage of proliferating cells, and the percentage reduction of alamarBlue fluorescent intensity as an index of cell viability. The EC_50_ (ng/mL) and the pEC_50_ (-log10, EC_50_, M) were determined using Hoechst as an index of the total number of cells. A non-linear curve was fitted using Graphpad Prism using the concentration of TNFa versus the relevant measure, normalised to vehicle (0.1% BSA/PBS, 0.01% DMSO). Data displayed is from four independent glioblastoma cases. Data represents mean ± SEM. ** = *P* < 0.01,  * = *P* < 0.05, ns = *P* > 0.05, of the IC_50_ or EC_50_ relative to vehicle + TNFa, or ## = *p* < 0.01 relative to TPCA-1 + TNFa (One-way ANOVA with Tukey’s multiple comparisons test)

## Discussion

One of the hallmarks of glioblastoma is the evasion of apoptosis. Therefore, identifying compounds that abrogate pro-survival signalling and sensitize cells to apoptosis has been recognized as an effective strategy towards the treatment of glioblastoma. Here, we identify a novel susceptibility of glioblastoma cells to TNFR1-dependent apoptosis, dependent on inhibition of canonical NFκB signalling using our previously reported palbociclib-HMCD conjugate, **1** [23]. Critically, **1** maintained the ability to inhibit nuclear pRb and E2F1 in patient-derived glioblastoma cells, and thus we can be confident that palbociclib was still acting on its CDK4/6 target when conjugated to MHI 148 (Fig. 3A).

Previously, we reported that conjugation of anti-cancer agents with HMCDs resulted in a significantly more potent compound by increasing the intracellular concentration of the compound as a result of uptake through transporters overexpressed on glioblastoma cells [15, 52]. Indeed, conjugation of cyclin-dependent kinase inhibitor, palbociclib, with an HMCD, MHI-148, resulted in a significantly more potent compound [1], improving the potency of anti-proliferative effects relative to palbociclib by more than 20-fold. Consistent with our previous studies on HMCD-drug conjugates [53–55], we expected conjugation of the cyclin-dependent kinase inhibitor, palbociclib would improve the anti-proliferative potency, relative to the palbociclib alone. However, we also reported a significant shift in the cytotoxicity of both palbociclib (94-fold, 352 ± 575 nM) when conjugated with MHI-148. This was particularly interesting as palbociclib did not induce any meaningful cytotoxic effects on patient-derived glioblastoma cells, with an EC_50_ of 30 ± 19 µM. Furthermore, we also demonstrated that **1** was significantly more potent than other FDA-approved cyclin-dependent kinase inhibitors across a range of patient-derived glioblastoma cell lines. Despite several studies exploring the synergism of CDK inhibitors with TMZ in glioblastoma, we reported an antagonistic response across all glioblastoma cell lines treated with palbociclib and TMZ. Whilst combining **1** with TMZ failed to further improve the potency, synergism with TMZ appeared to be case-dependent, independent of MGMT methylation status, and only occurred at higher effect sizes. Hence, given the concentrations of TMZ achieved in glioblastoma tissue, (0.6 µg/mL, 3.08 µM) following oral administration of a single dose of TMZ (150 mg/kg^2^) [56, 57], it is unlikely a synergistic response will be achieved with therapeutically relevant doses.

Multiple preclinical studies have indicated that CDK4/6 inhibitors exhibit a synergistic effect with radiation both in vitro and in vivo [58–61]. Whilst several small sample clinical studies have indicated that the use of CDK4/6 inhibitors were well tolerated in adult and paediatric high-grade glioma, to the best of our knowledge, the combination with radiotherapy has not been explored clinically [13, 59, 62, 63]. Further work is required to explore optimal radiotherapy-drug combinations, given the success of radiotherapy in the treatment of glioblastoma. Despite the lack of synergism of **1** with TMZ, we demonstrated strong radiation-sensitization effects of **1** with a single 3 Gy dose of radiation across all modalities investigated, including increased γH2AX foci, reduced RAD51 expression and, consequently, greater apoptosis. Whilst it was encouraging that the radiation-sensitization effects of palbociclib were maintained in **1**, further work should investigate the effects of these compounds on radiation-resistant models to establish whether their efficacy is maintained in models following the standard of care.

Interestingly, our investigation showed manifested that conjugation of palbociclib to MHI-148 resulted in TNFR1 upregulation and the release of endogenous TNFα from glioblastoma cells. Collectively, these events resulted in alterations in the functional state of the canonical TNFα pathway from NFκB-dependent cell survival to death-inducing complex formation. This was evidenced by the inhibition of TNFα-induced NFκB signalling events. In addition, the importance of TNFR1 in **1**-induced apoptosis was further supported by TNFR1 knockdown and inhibition of TNFR1 endocytosis abrogating glioblastoma cell apoptosis. Interestingly, recent work by Debnath et al., 2022 further affirmed the TNFR1-cell death pathway as a targetable axis in peripheral cancer cells with the flavonoid, eritodictyol [64]. This pathway was also implicated in the sensitization of glioblastoma cells to TNFα through the NFκB inhibitor, Ebselen [35], and NFκB-dependent TMZ-sensitization through IAP inhibitor BV6 [65]. However, our study demonstrated for the first time that inhibition of NFκB and concurrent activation of TNFα-TNFR1 signalling results in potent cytotoxicity in patient-derived glioblastoma cells.

Further investigation revealed that **1** induced TNFR1-dependent extrinsic apoptosis in glioblastoma cells, as evident from the expression profile of hallmark apoptosis-related proteins. The approach of selectively targeting the extrinsic route to induce apoptosis in glioblastoma cells, such as through ligation of death receptors, is appealing for cancer therapy, since death receptors have a direct link to the cell’s death machinery. In brief, we demonstrated that exposure of **1** to glioblastoma cells caused time and concentration-dependent upregulation of endogenous TNFα and TNFR1 receptor. Upregulation of TNFR1 was dependent on the delivery of newly synthesised TNFR1 to the plasma membrane, as evidenced by a reduction in the phenotype in response to BFA and CHX inhibition. BFA treatment increased the total expression of TNFR1, supporting the notion that **1** may have a chaperone effect on TNFR1, stabilising new receptors and preventing cleavage from the plasma membrane. Consequently, we reported an increase in the transcription and expression of the DISC complex components, including TNFR1, FADD and TRADD, leading to the activation of cleaved caspase 8 and ultimately resulting in caspase-dependent cell death of glioblastoma cells.

This study describes several important points. First is the discovery that CDK4/6-dependent cell death is driven by the increase in expression of TNFR1 and the concurrent inhibition of the NFκB pathway. This knowledge may suggest potential therapeutic targets that could be exploited by other compounds that have similar mechanisms of action. For example, anti-inflammatory compounds, such as ibuprofen, aspirin and indomethacin, which are known inhibitors of NFκB [66–69] could be explored in combination with pro-apoptotic agents, or rhTNFα, or other ligands of TNFR1 for glioblastoma treatment. Secondly, this study suggests that new, undiscovered mechanisms of action of our conjugate may exist. It has become increasingly apparent that kinase inhibitors developed to target a particular signalling pathway are likely having more wide-spread effects on other intracellular targets [31, 32, 70], which could be leveraged to expand the therapeutic indices of existing compounds without invoking unnecessary toxicity. Furthermore, it is likely that many therapies like palbociclib may alter the apoptotic threshold as a single agent but may not be capable of triggering cell death alone [64]. Hence, this study further affirms there is a great deal of interest in leveraging kinase inhibitors to enhance the efficacy of immune-based approaches, such as rhTNFα treatment for glioblastoma patients. Understanding the mechanisms that determine the fate of TNFα signalling may provide the opportunity to identify novel therapeutic targets to sensitize glioblastoma tumours to TNFα treatment. Ultimately, we reported that conjugation of palbociclib to MHI 148 results in a potent compound that synergizes with TNFα to promote TNFα-TNFR1-dependent cell death.

## Conclusion

Ultimately, this work identified a novel susceptibility of glioblastoma cells to TNFR1-dependent apoptosis, dependent on inhibition of canonical NFκB signalling using our previously reported palbociclib-HMCD conjugate, 1 [23]. We show that patient-derived glioblastoma cells are capable of autocrine/paracrine TNFα-TNFR1 dependent cell death, hence identifying TNFR1, TNFα and NFκB as key targets in this pathway, future work could use drug libraries in order to uncover novel TNFα-sensitising or cytotoxic agents for use in glioblastoma. Given the pleiotropic effects of TNFα in the brain, identifying compounds that induce localised TNFα secretion and pro-apoptotic TNFR1 signalling provides a more compelling argument for TNFα-based therapies. However, future work should investigate the effects of 1-induced TNFα-TNFR1-dependent cell death in a more complex in vitro or in vivo model.

### Supplementary Information

**Additional file 1: Supplementary Figure 1.** Cyclin-dependent kinase inhibitor-MHI-148 conjugates, 1 are not synergistic with TMZ. Patient-derived glioblastoma cells were treated with up to 100 μM of palbociclib, and 1 with and without 100 μM of TMZ for 96 h. Concentration-dependent effects of each compound on the toxicity of glioblastoma cells was measured by the percentage of Hoechst-positive cells after 96 h (EC_50_). A non-linear curve was fitted using Graphpad Prism using the concentration of each compound versus the percentage of hoechst-positive cells (A). Combination index (CI) was calculated from the CI equation algorithms using CompuSyn software. CI=1, <1 and >1 indicates additive effect, synergism, and antagonism, respectively (B). The pEC50 of each compound with and without TMZ (100 μM) on each glioblastoma cell line is summarised in B and D. The CI for palbociclib (A) and 1 (C), with TMZ across a range of effect sizes is presented per case. Data represents mean ±SEM for at least six independent glioblastoma cases, ns = *p* > 0.05. **Supplementary Figure 2.** Trafficking and expression of TNFR1 in response to 1 in patient-derived glioblastoma cells in the presence of vesicle trafficking inhibitor, BFA and protein translation inhibitor, CHX. Residual starting surface expression TNFR1 (Method A) (A). To investigate the net surface expression of TNFR1 (Method B) (B). C summarises method A and B detection of TNFR1. Total TNFR1 expression in response to 1 in the presence of absence of BFA or CHX (D) Summary data of the pEC_50_ (E) and E_max_ (F) of 1 on the total TNFR1 expression in the presence or absence of BFA or CHX. Basal total TNFR1 expression in G. Data represents mean ± SEM from three independent glioblastoma cases. ns = *p* > 0.05, * *p* < 0.05, ** *p*< 0.01, *** *p* < 0.001, One-way ANOVA relative to 1 plus vehicle inhibitor.

## Data Availability

Additional data could be available on request from the authors.
