# Supplementary material for: Involvement of the tumour necrosis factor receptor system in glioblastoma cell death induced by palbociclib-heptamethine cyanine dye conjugate
Source: Cell Commun Signal. 2024 Jan 11;22:30. doi: 10.1186/s12964-023-01277-z (PMC10782607; doi:10.1186/s12964-023-01277-z)
Supplement: Supplementary file 2 — Additional file 1: Supplementary Figure 1. Cyclin-dependent kinase inhibitor-MHI-148 conjugates, 1 are not synergistic with TMZ. Patient-derived glioblastoma cells were treated with up to 100 μM of palbociclib, and 1 with and without 100 μM of TMZ for 96 h. Concentration-dependent effects of each compound on the toxicity of glioblastoma cells was measured by the percentage of Hoechst-positive cells after 96 h (EC50). A non-linear curve was fitted using Graphpad Prism using the concentration of each compound versus the percentage of hoechst-positive cells (A). Combination index (CI) was calculated from the CI equation algorithms using CompuSyn software. CI=1, <1 and >1 indicates additive effect, synergism, and antagonism, respectively (B). The pEC50 of each compound with and without TMZ (100 μM) on each glioblastoma cell line is summarised in B and D. The CI for palbociclib (A) and 1 (C), with TMZ across a range of effect sizes is presented per case. Data represents mean ±SEM for at least six independent glioblastoma cases, ns = p > 0.05. Supplementary Figure 2. Trafficking and expression of TNFR1 in response to 1 in patient-derived glioblastoma cells in the presence of vesicle trafficking inhibitor, BFA and protein translation inhibitor, CHX. Residual starting surface expression TNFR1 (Method A) (A). To investigate the net surface expression of TNFR1 (Method B) (B). C summarises method A and B detection of TNFR1. Total TNFR1 expression in response to 1 in the presence of absence of BFA or CHX (D) Summary data of the pEC50 (E) and Emax (F) of 1 on the total TNFR1 expression in the presence or absence of BFA or CHX. Basal total TNFR1 expression in G. Data represents mean ± SEM from three independent glioblastoma cases. ns = p > 0.05, * p < 0.05, ** p< 0.01, *** p < 0.001, One-way ANOVA relative to 1 plus vehicle inhibitor. [file 12964_2023_1277_MOESM1_ESM.docx]

# Supplementary Figures


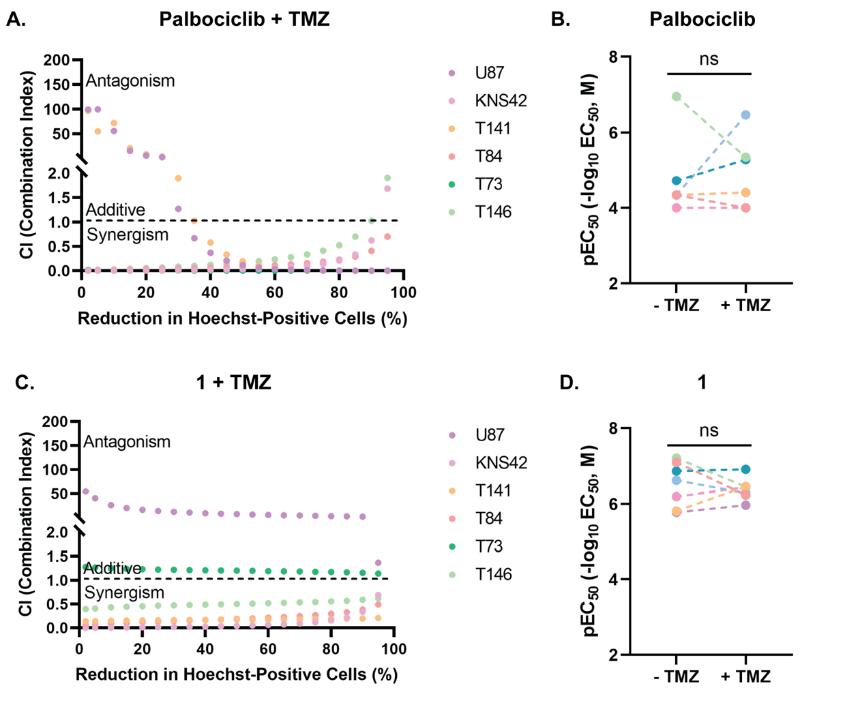


Supplementary Figure 1. Cyclin-dependent kinase inhibitor-MHI-148 conjugates, 1 are not synergistic with TMZ. Patient-derived glioblastoma cells were treated with up to 100 μM of palbociclib, and 1 with and without 100 μM of TMZ for 96 h. Concentration-dependent effects of each compound on the toxicity of glioblastoma cells was measured by the percentage of Hoechst-positive cells after 96 h (EC_50_). A non-linear curve was fitted using Graphpad Prism using the concentration of each compound versus the percentage of hoechst-positive cells (A). Combination index (CI) was calculated from the CI equation algorithms using CompuSyn software. CI=1, <1 and >1 indicates additive effect, synergism, and antagonism, respectively (B). The pEC50 of each compound with and without TMZ (100 μM) on each glioblastoma cell line is summarised in B and D. The CI for palbociclib (A) and 1 (C), with TMZ across a range of effect sizes is presented per case. Data represents mean ±SEM for at least six independent glioblastoma cases, ns = p > 0.05.


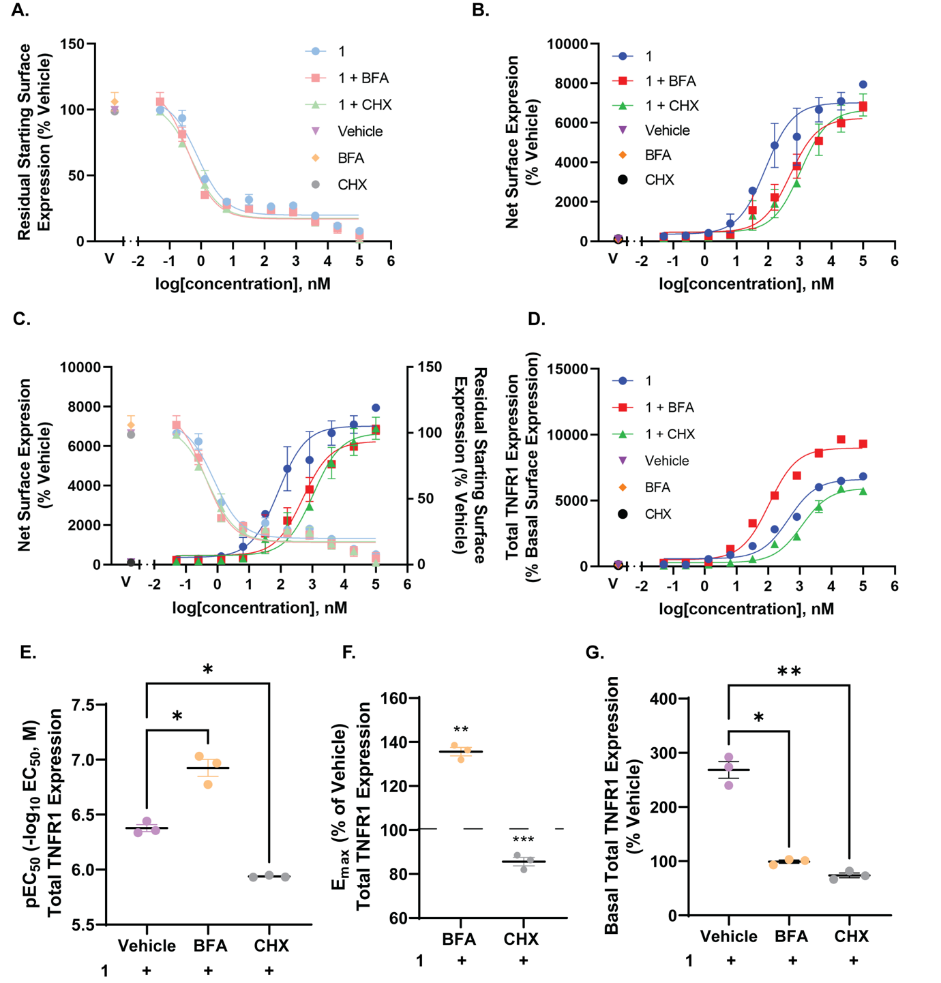


Supplementary Figure 2 Trafficking and expression of TNFR1 in response to 1 in patient-derived glioblastoma cells in the presence of vesicle trafficking inhibitor, BFA and protein translation inhibitor, CHX

Residual starting surface expression TNFR1 (Method A) (A). To investigate the net surface expression of TNFR1 (Method B) (B). C summarises method A and B detection of TNFR1. Total TNFR1 expression in response to **1** in the presence of absence of BFA or CHX (D) Summary data of the pEC_50_ (E) and E_max_ (F) of **1** on the total TNFR1 expression in the presence or absence of BFA or CHX. Basal total TNFR1 expression in G. Data represents mean ± SEM from three independent glioblastoma cases. ns = p > 0.05, * *p* < 0.05, ** *p* < 0.01, *** *p* < 0.001, One-way ANOVA relative to **1** plus vehicle inhibitor.
